# It Is Time to Get to Know the Neuroendocrine Cell Hyperplasia of Infancy

**DOI:** 10.1111/crj.13827

**Published:** 2024-08-13

**Authors:** Long Jin, Wen Wei

**Affiliations:** ^1^ Department of Respiratory Medicine Anhui Provincial Children's Hospital Hefei Anhui China

**Keywords:** children, interstitial lung disease, neuroendocrine cell hyperplasia of infancy, review

## Abstract

In the two decades that have elapsed since the initial proposal of neuroendocrine cell hyperplasia of infancy (NEHI), several hundred cases have been reported and researched. However, a comprehensive analysis of research progress remains absent from the literature. The present article endeavors to evaluate the current progress of NEHI research and offer a reference for the clinical management of this condition.

## Graphical abstract

Neuroendocrine cell hyperplasia of infancy (NEHI) is a common form of childhood interstitial lung disease. A high‐resolution CT scan is a useful and major imaging approach for assisting in diagnosis. The major characteristics of NEHI on CT are the presence of typical ground glass opacities and air trapping. An accurate diagnosis is the key to guiding appropriate clinical management.

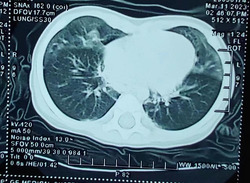

## 1 Background

Neuroendocrine cell (NEC) hyperplasia of infancy (NEHI), initially identified as persistent shortness of breath in infancy [1], was first reported by Professor Deterding et al. [2] in 2005. This disorder represents an interstitial and diffuse lung disease with an indeterminate etiology in pediatric populations [3] and is named following the observation of increased NECs in the airways in biopsy. Clinical manifestations include tachypnea, persistent shortness of breath, retractions, crackles in the lungs, and hypoxemia [2, 4]. NEHI predominantly occurs in infants within their first 2 years of life, with a mean age of 3 months, and most patients are born full‐term [5, 6]. To date, only one premature infant case has been documented [7].

The clinical manifestations of NEHI frequently resemble other interstitial pulmonary disorders, resulting in a substantially lower number of reported NEHI cases than its actual prevalence, which is likely due to the minimal need for lung biopsies in patients with mild conditions and the general lack of knowledge regarding the disease [8]. The nonspecific nature of its clinical features, together with the limited number of previously documented cases, contribute to the lack of experience in clinical management. Pediatric respiratory specialists face challenges in accurately identifying, diagnosing, and treating NEHI, and this would potentially lead to underdiagnosis, misdiagnosis, and delay in the treatment of NEHI in children. Against this background, it is crucial to visit the latest evidence for the diagnosis, treatment, and prognosis of NEHI in pediatric populations to enhance our understanding of the disease per se and its management. This review will provide a literature review by summarizing the existing evidence for NEHI to inform evidence‐based clinical practice and enhance pediatric clinicians' capacity to accurately identify, diagnose, and properly treat children with NEHI.

## 2 Pathophysiological Features of NEHI

### 2.1 Pulmonary NECs

**FIGURE 1:**
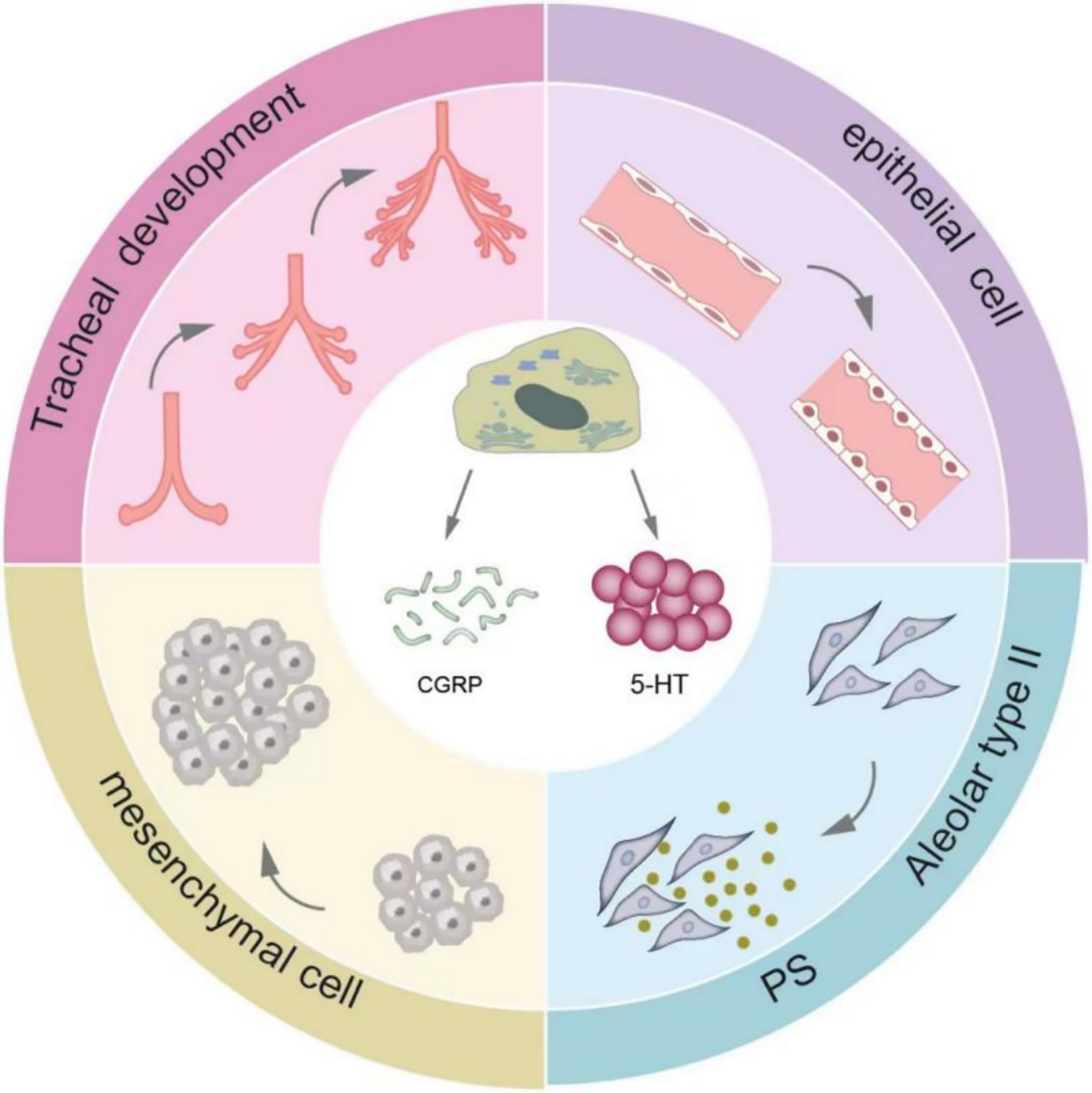
Physiological role of pulmonary neuroendocrine cells. Legend: Pulmonary neuroendocrine cells (NECs) are mainly distributed within the distal airways, contributing to tracheal development, mesenchymal and epithelial cell proliferation, and the secretion of alveolar surfactant substances to further produce pulmonary surfactant (PS). Hyperactive pulmonary NECs can produce abundant calcitonin gene‐related peptide (CGRP) and 5‐hydroxytryptamine (5‐HT).

**FIGURE 2:**
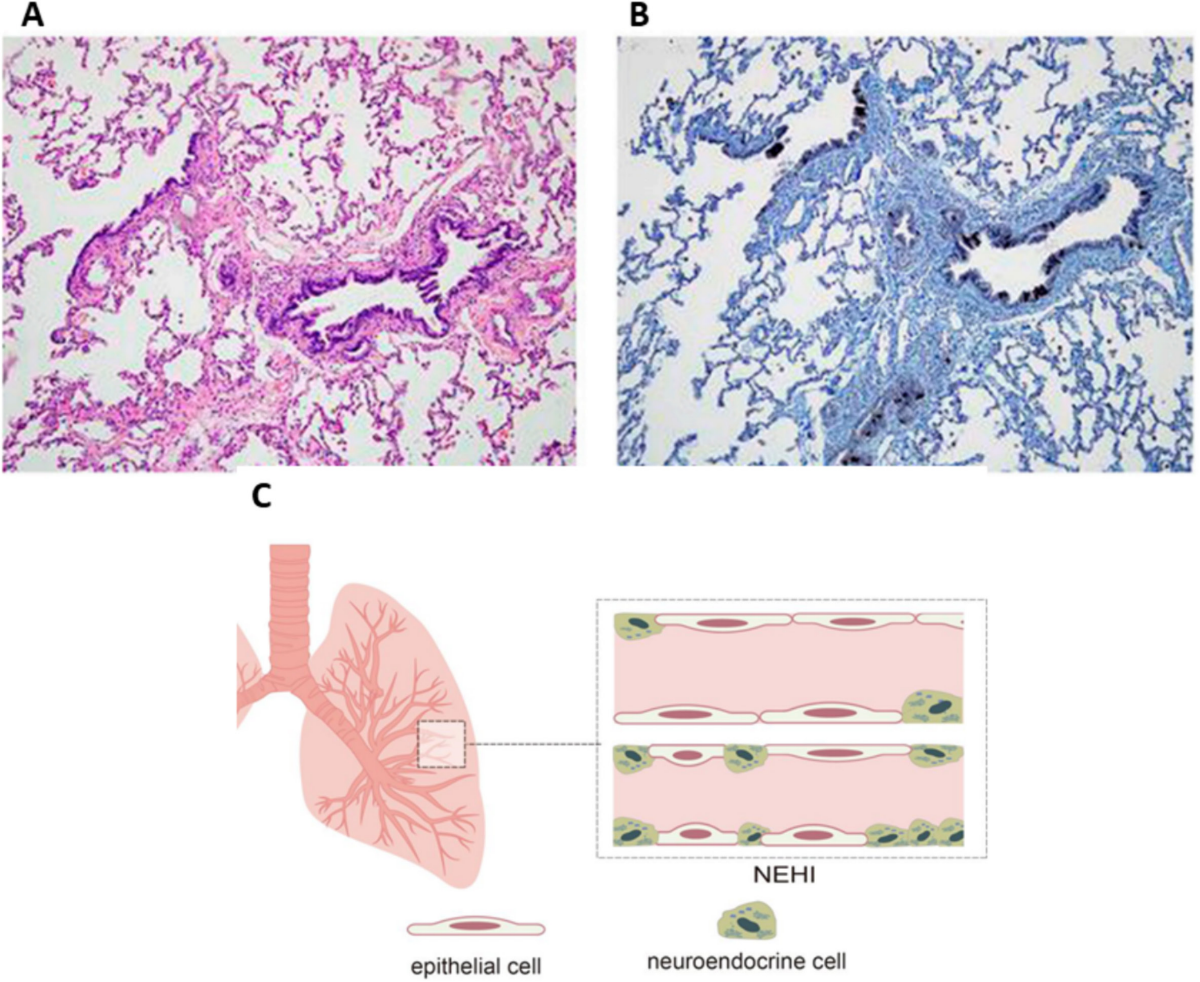
Histologic features of neuroendocrine cell hyperplasia of infancy (NEHI). Legend: (A) and (B) are figures reused from the paper by Popler et al. [9] with permission. (A) (HE staining) and (B) (bombesin immunohistochemistry) show the histologic features of NEHI, including significant inflammation of fibrosis of the bronchioles on hematoxylin and eosin (HE) staining and increased bombesin‐positive airway neuroendocrine cells (NECs) in the respiratory bronchioles and alveolar ducts. (C) illustrates a large number of neuroendocrine cells in the lung tissue of children with NEHI compared to normal children.

The histology of NEHI is marked by increased pulmonary NECs in the airway epithelium and lobular parenchyma and an absence of notable interstitial inflammation [2, 4]. Pulmonary NECs are granular epithelial cells distributed in distal airways, predominantly contributing to branching morphogenesis, mesenchymal and epithelial cell proliferation, and the secretion of alveolar surfactant substances. NECs also induce the proliferation of airway epithelial and mesenchymal cells, as well as the differentiation of alveolar Type II cells (Figure 1). There is a large amount of pulmonary NECs in the fetal and neonatal periods, but the number rapidly decreases in the first year of life [2]. In NEHI pediatric patients, hematoxylin and eosin staining shows bronchiolar inflammation and fibrosis (Figure 2A) and bombesin immunohistochemistry found increased bombesin‐positive airway NECs of respiratory bronchioles and alveolar ducts (Figure 2B,C) [9, 10].

### 2.2 Roles of Calcitonin Gene‐Related Peptides (CGRPs) and 5‐Hydroxytryptamine (5‐HT)

**FIGURE 3:**
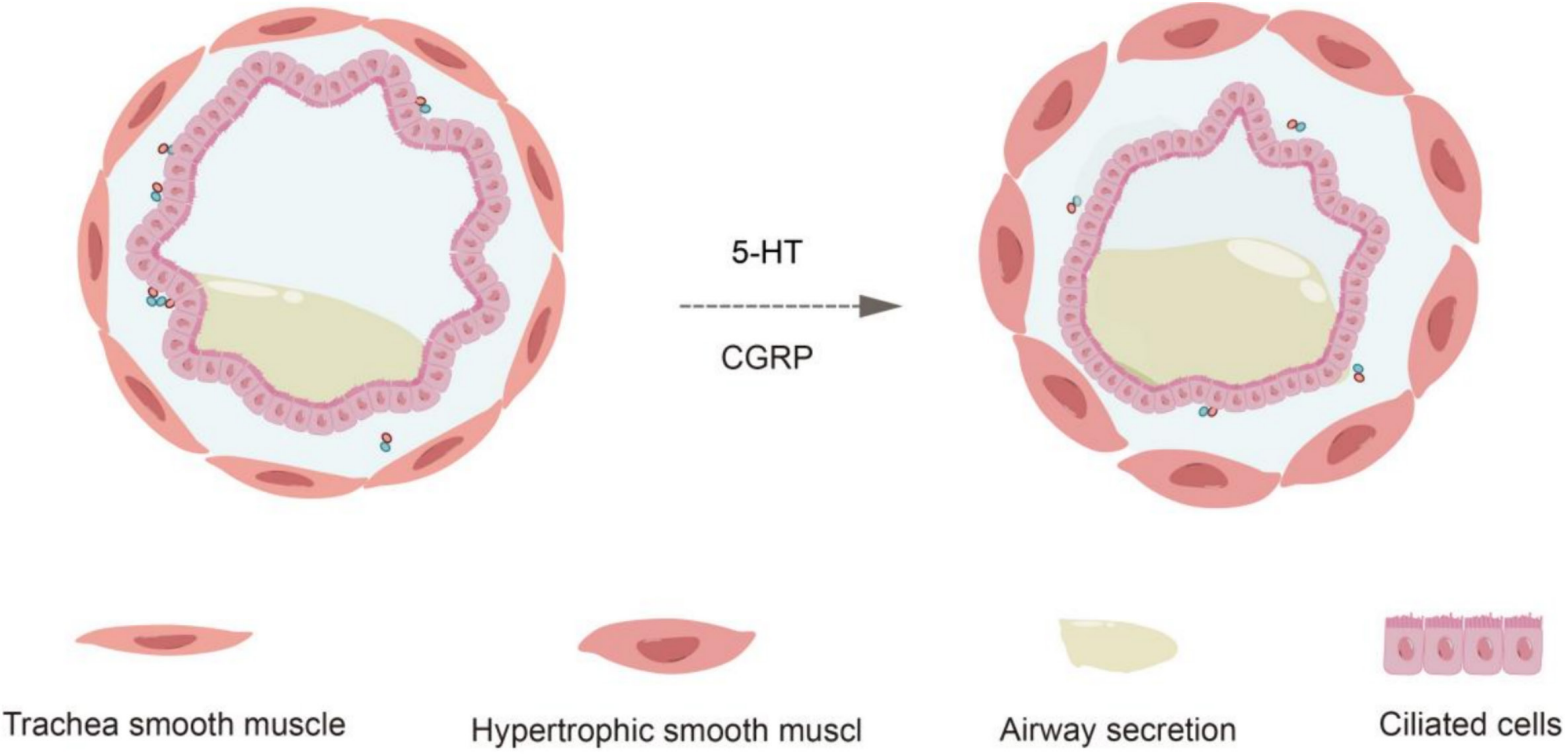
Pathological changes caused by neuroendocrine cells through 5‐HT and CGRP.

Hyperactive pulmonary NECs can produce copious amounts of CGRP and 5‐HT, which, in turn, stimulate a cascade of abnormal physiological changes by modulating bronchial smooth muscle tone, local immune responses in the lung, pulmonary blood flow distribution, as well as stimulation of sensory nerve fibers in the lung and regulation of lung growth and development [9, 11, 12, 13, 14, 15]. Yang et al. [16] corroborated the involvement of 5‐HT in the inflammatory response to obstructive lung lesions through mouse experiments. CGRP has been shown to increase endothelium permeability and lead to excessive lung fluid and hypoxemia [17, 18, 19]. Additionally, CGRP can lead to bronchial smooth muscle contraction and regulate local immune responses in the lung via various immunomodulatory effects (Figure 3) [20, 21, 22, 23]. In several studies, CGRP was found to stimulate IL‐5 production by innate lymphoid cells (ILC2), and an increase in its receptor Calcrl can lead to a rise in Th2 cells and eosinophils following allergen stimulation [24, 25]. The process of pathophysiological alterations eventually results in air stagnation and small airway obstruction, which, subsequently, induce hypoxemia in infants and a spectrum of clinical symptoms.

## 3 Causes of NEHI

The underlying cause of the elevated and overexpressed NECs in the lungs and NEHI development remains unclear. Several studies have revealed that neither pulmonary infections nor environmental toxins are implicated in the disease's development [7, 8, 26, 27]. Others reported that acute respiratory viral infections might be implicated in the pathogenesis of NEHI [28]. This was supported by a study observing the presence of the Epstein–Barr virus in the peribronchial lymphocytes of children with NEHI [13].

In 2010, Popler et al. published a report on a case series of NEHI involving siblings from four distinct families, all of whom were diagnosed with NEHI, highlighting the crucial role of genetic factors in NEHI pathogenesis [29]. Young et al. [10] detected a heterozygous substitution in the NKX2.1 gene, which encodes TTF‐1 (also referred to as Thyroid Transcription Factor 1), in families with a history of NEHI in childhood. However, later research indicated that NKX2.1 mutations were associated with the “brain‐thyroid‐pulmonary” syndrome and a variety of more severe pulmonary phenotypes. NKX2.1 mutations may contribute, but they are not the primary cause of NEHI. Aberrant expression of NKX2.1 target genes may be responsible for the pulmonary pathophysiology of NEHI. In 2016, Berteloot, Galmiche‐Rolland, and Abou‐Taam [30] reported one case of a single mutation in the ABCA3 gene in a child clinically diagnosed with NEHI, identified through gene sequencing during follow‐up. Nevertheless, none of the existing reports have been able to pinpoint the causative gene of NEHI.

## 4 Clinical Manifestations of NEHI

### 4.1 Pulmonary Signs and Symptoms

NEHI typically develops within the first year of life. The median age of symptom onset is 3 months old, whereas a formal diagnosis is typically given at 6 months of age [5]. The most prevalent pulmonary signs and symptoms of NEHI include tachypnea, shortness of breath, hypoxemia (> 90%), and pulmonary crackles (> 80%). More severely, NEHI can cause intercostal retractions, which can be aggravated by acute viral infections [5, 28]. In addition, acropachy, cough, and/or gasp are also seen in NEHI patients [28, 31, 32, 33]. Pediatric patients with NEHI are generally clinically stable, and the disease is often self‐limited, with respiratory failure rarely reported.

### 4.2 Extrapulmonary Manifestations and Complications

Failure to thrive and developmental delay over the long term are common extrapulmonary signs, primarily attributed to long‐standing systemic hypoxia, recurrent hospitalizations, and undertreatment resulting from misdiagnosis and a lack of experience in disease management [32]. Other complications of NEHI have also been documented. According to a study of 199 children diagnosed with NEHI, more than half of the children had dysplasia, 101 of them had gastroesophageal reflux, 75 of them suffered from aspiration or were at risk of aspiration, and 34 of them demonstrated signs of abnormalities associated with the immune system [31]. The gastroesophageal reflux may be attributed to increased elastic resistance in the lungs due to hyperinflation, which subsequently results in heightened negative intrapleural pressure. This higher negative intrathoracic pressure then leads to a diminished pressure gradient from the stomach to the intrathoracic esophagus, causing an increase in reflux [34, 35, 36]. A significant proportion of patients with NEHI experience misaspiration because of difficult breathing and increased respiratory load, which hinders respiratory‐swallowing coordination. Besides these conditions, it was documented that a few NEHI patients also exhibited transient hypogammaglobulinemia [31, 37], characterized by elevated IgA, IgE, IgG, and IgM and decreased IgA and Complement 3, although the underlying mechanism remains unclear.

## 5 Diagnosis

### 5.1 The Gold Standard for Diagnosing NEHI

Lung biopsies served as a gold standard for NEHI diagnosis [38]. However, the invasive nature of lung biopsies and the associated procedural challenges contribute to their practical difficulty. In a 2011 study by Young et al., it was demonstrated that the increased presence of NECs in NEHI was not specific compared to other lung injuries or diseases, and diagnosis still requires a combination of clinical, radiological, and pathological assessments [10]. With a deeper understanding of the disease, an increasing number of scholars contend that lung biopsy‐based NEHI diagnosis should be gradually replaced by a clinical and radiological diagnostic approach assessing clinical significance, sociological aspects, invasiveness, and acceptance by the child's family [5, 39, 40, 41].

### 5.2 Radiological Findings

**FIGURE 4:**
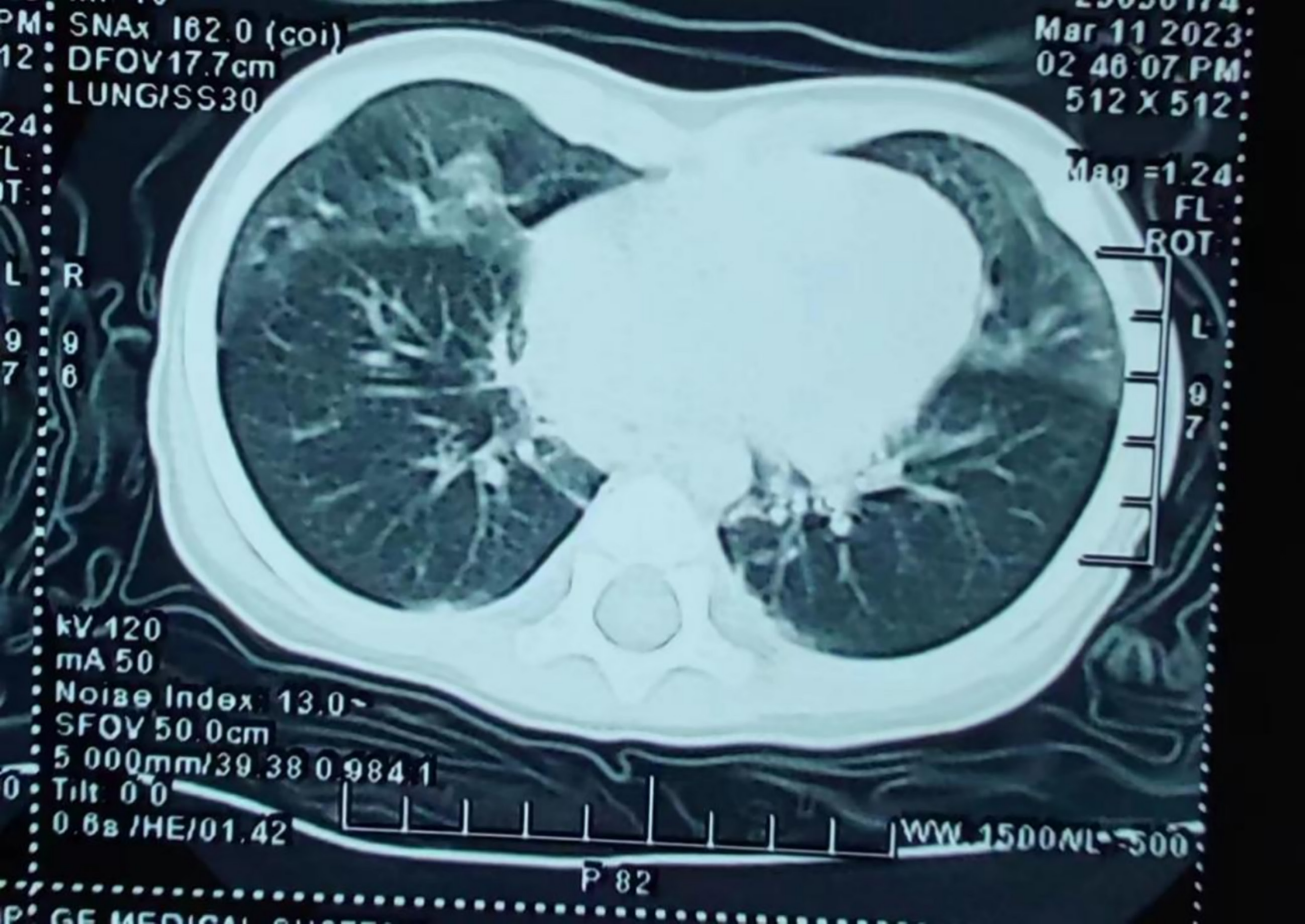
Typical ground glass opacities and air retention on chest CT of children with NEHI.

Imaging studies have shown that NEHI can be identified on high‐resolution computed tomography (HRCT) by characteristic air trapping presenting in a mosaic pattern and ground glass opacities (GGOs) primarily occurring in the right middle lobe, lingula, and/or perihilar regions (Figure 4) [8, 40, 42, 43, 44, 45]. The CT images of 23 children with biopsy‐confirmed NEHI were evaluated without any clinical information from a radiologist, and six CT images of children with other interstitial lung diseases (ILDs) were included for comparison [42]. According to the study, CT imaging was capable of diagnosing NEHI with a sensitivity and specificity of at least 78% and 100%, respectively. Using a specific Hounsfield unit threshold, Spielberg [43] quantified GGO in 21 children with NEHI and investigated their HRCTs. The study demonstrated that GGO was predominant in the right middle and lingual lobes in subjects with NEHI and that it was more prevalent in children who required continuous oxygen as opposed to those who only received oxygen at night, suggesting that the size of the GGO correlates with the severity of the child's hypoxic symptoms.

Mastej et al. [44] studied the airway and lung shape in children with NEHI compared to children in the control group in a series of studies and revealed statistical differences between the two groups, with children diagnosed with NEHI exhibiting wider apices and significantly larger anteroposterior diameters of the lung apex. The use of logistic regression models to differentiate children with NEHI from children without NEHI showed an accuracy rate of up to 90%. Additionally, Barrera et al. [45] utilized quantitative chest CT to diagnose NEHI and found that patients with this condition had a lower mean lung density, a higher ventilation heterogeneity, an increased lung mass, and a larger lung volume.

### 5.3 Lung Function Test and Ultrasound Findings

Over recent years, there has been an increased use of lung function testing and lung ultrasound to aid in diagnosing NEHI. A significant degree of air retention was found in children with NEHI that is not directly related to the degrees of obstructive lesions [13, 15, 46]. The results of an infant pulmonary function test (iPFT) with an FRCpleth of 150% of the predicted value have also been shown to be highly specific for diagnosing NEHI and may aid in the detection of the disease at an early stage. Lung ultrasound was also found to be highly sensitive, albeit nonspecific, in diagnosing children with NEHI [47]. Children with NEHI were found to have increased numbers and density of B‐lines, as well as pleural thickness and irregularity. Nonetheless, if GGO is centrally located, the ultrasound results may appear normal. It is worth noting that increased B‐lines can also be seen in other lung diseases such as pulmonary edema, respiratory tract infections, and atelectasis. Similarly, pleural thickness is commonly seen in pleural diseases such as pleuritis. In the future, lung ultrasound may become a useful initial evaluation for NEHI diagnosis in children suspected of NEHI due to its simplicity, noninvasive nature, and lack of radiation before performing HRCT, which is associated with the risk of radiation exposure and is costly.

### 5.4 Polysomnography (PSG)

Previous studies also found that PSG is useful for assisting clinicians in differentiating NEHI from other lung diseases in pediatric patients. Among 14 children diagnosed with NEHI, PSG found that patients with NEHI were likely to experience sleep‐related breathing disorders, such as obstructive or central sleep apnea, hypoxemia, adenoidal/tonsillar hypertrophy, low sleep efficiency, and increased periodic limbic dyskinesia [48]. Despite not being routinely recommended, PSG may be useful for pediatric patients with NEHI who also present with sleep‐related disorders. PSG will also be helpful in determining the need for prolonged oxygen therapy during the night and monitoring sleep‐related breathing diseases in children with NEHI.

### 5.5 Risk Scores for NEHI

In 2020, Karpenko et al. conducted a multicenter study involving 83 patients with NEHI and proposed a 10‐point scale for the diagnosis of NEHI [49]. The scale is comprised of scores for various symptoms such as chest wall depression, immune abnormality (low immunoglobulin G), hypoxemia, shortness of breath, dysplasia, chest burst sound, activity intolerance, irregular cough, and irregular wheeze, with each item being scored as 1 point. The proposed scale sets a score greater than 7 points as a diagnostic criterion for NEHI, with a sensitivity of 87%. The study's sample size is relatively large and provides a high reference value for the diagnosis of the disease using a scale‐based approach. The author recommends the inclusion of complications such as gastroesophageal reflux and dysphagia reported in recent years, along with clinical auxiliary examinations such as CT, lung function, lung ultrasound, and sleep monitoring, coupled with an adjustment of the scoring rules relative to the clinical manifestations and diagnostic significance of each auxiliary examination, potentially leading to a more effective diagnosis of NEHI. A more recent study found that, by using this scoring algorithm, only around two‐thirds of NEHI cases reached Score 7 or higher [50]. They recommend removing less pertinent criteria to improve the discrimination ability of this scoring. More studies are needed to validate this scoring algorithm and identify the best scoring approach with both high sensitivity and specificity.

### 5.6 Differentiating NEHI From Other Child ILDs

Given that NEHI is one form of childhood ILD and corticosteroids are not effective in treating NEHI, differentiating NEHI from other ILDs and common pediatric respiratory diseases is important for guiding appropriate treatments in clinical practice [29]. Pulmonary interstitial glycogenosis (PIG), surfactant dysfunction disorders, and alveolar capillary dysplasia with misalignment of pulmonary veins are other ILDs commonly seen in pediatric populations. The first step in the NEHI investigation is to exclude non‐ILD diseases. To trigger the investigation of childhood ILD, at least three of the following four criteria have to be met [51]. The first is the presence of any respiratory symptoms, including cough, tachypnoea, and/or not being able to tolerate exercise; the second is clinical signs, which include resting tachypnoea, adventitious sounds, retraction, finger clubbing, failure to thrive, and aggressive respiratory failure. The third is the presentation of hypoxemia, and the last is the diffuse abnormalities on chest X‐rays or CT scanning. For the specific types of ILD, symptoms of PIG and surfactant dysfunction disorder more commonly occur shortly after birth. Alveolar capillary dysplasia with misalignment of pulmonary veins is more commonly seen in those with congenital abnormalities. In addition to the common lung function, full blood testing, and ECG, an echocardiogram is recommended to preclude any structural cardiovascular disease and pulmonary hypertension, which account for approximately 9% of pediatric patients suspected of ILD.

A lung biopsy is useful for the diagnosis of most ILDs. For example, PIG can be diagnosed via the observation of histological changes, including the presence of a circle of glycogen‐laden mesenchymal cells that widen the interstitial walls. However, one must be vigilant when using a lung biopsy due to the potential risk of this invasive procedure. Therefore, noninvasive techniques in combination with age at presentation, clinical manifestations, and consistent pulmonary function presentation would be useful in diagnosing the disease. Chest X‐ray, ultrasound, and pulmonary function tests may serve as initial evaluations to exclude other common entities of ILD, such as cystic fibrosis and infection, before HRCT. In the suspected NEHI cases, an HRCT scan with the lowest radiation dose can provide accurate information. Two studies [27, 52] also reported that bronchoscopy with bronchoalveolar lavage (BAL) cellular analysis can differentiate NEHI from other types of ILD and lung diseases (e.g., cystic fibrosis and follicular bronchiolitis) in pediatric patients. In Deterding et al.'s study [27], children with NEHI had more aptamers in BAL fluid compared to those with other ILDs. Conversely, proteins associated with pulmonary fibrosis and inflammation were found in children with surfactant dysfunction mutations but not in NEHI patients. NHEI patients were also found to have lower total white blood cells, IL‐1β, MIP‐1β, and IL‐8 and higher alveolar macrophages in BAL fluid compared with patients with other airway diseases [52]. These findings need to be confirmed by more studies. In addition, BAL analysis can also be used to rule out infection, aspiration, pulmonary hemorrhage syndromes, and pulmonary alveolar proteinosis [53]. Genetic tests can be performed if genetic mutations and family inheritance are suspected, which were assumed to be the reasons for NEHI [29, 54]. A lung biopsy with immunostaining for bombesin is only needed when other tests will not be able to give a precise diagnosis, and the diagnosis is necessarily needed for guiding disease management [53].

## 6 Treatment and Prognosis

### 6.1 Treatment for NEHI

The progression of NEHI is slow and prolonged, and at this point, there have been no reports of fatalities. Despite extensive research, there is no specific treatment available for NEHI. Previous studies and empirical evidence showed that antibiotic and glucocorticoid treatments provide little benefit in treating NEHI [7, 8, 13]. Therefore, the treatment principles involve supportive care and infection prevention. Oxygen supplement is the cornerstone of supportive therapy, which can alleviate hypoxemia and improve breath difficulty. Of note, it is important to monitor the patient regularly with nocturnal saturation recording to adapt to oxygen therapy. In clinical practice, the degree of hypoxia in children varies: most children with NEHI are asymptomatic at rest and require oxygen supplementation only after exercise or during respiratory infections, while a few children require long‐term oxygen therapy with a proactive approach taken to prevent complications such as gastroesophageal reflux and misaspiration.

Because of the rarity and heterogeneity of NEHI and other child ILDs, there is intrinsic difficulty in conducting randomized trials to test the efficacy of new treatments in this patient group. The current therapeutic strategies for NEHI are empirical and mainly based on case series. A recent Phase 2a, double‐blind randomized clinical trial comparing hydroxychloroquine versus placebo in 26 pediatric patients with ILD found no noticeable benefits of hydroxychloroquine in improving O_2_ saturation, pulmonary function, and quality of life [55]. Gene transfer therapies have shown some promise in treating child ILD in vitro and in vivo. Clinical studies are needed to assess whether gene transfer confers any benefits to NEHI cases with specific gene mutations [56].

### 6.2 Prognosis of NEHI

In terms of prognosis, it is recommended that gamma globulin levels be routinely assessed in patients with NEHI in order to monitor their risk of respiratory infections, and prophylactic antibiotic therapy and/or intravenous immunoglobulin should be administered if necessary [31]. In addition, many patients with NEHI require nutritional supplementation when presenting difficulty feeding and poor weight gain. Pneumococcal and influenza vaccines are recommended for NEHI patients at 6 months of age to prevent pneumonia and other infections [8]. Avoiding exposure to detrimental environments, for example, second‐hand smoke, and keeping good hygiene for children and their parents are also important [57]. In the early years, NEHI was often misdiagnosed as a pulmonary infection or other ILDs, leading to the administration of unnecessary empiric medications such as antibiotics and corticosteroids. Such treatments hurt the child's immune system and adversely impact their physical and mental health. However, clinical treatment for NEHI is typically straightforward with a precise diagnosis.

In general, NEHI offers a favorable prognosis. The majority of children in multicenter case follow‐ups were no longer dependent on oxygen supplementation by the age of 2 years (20–60 months), and their clinical symptoms had disappeared by that time. However, some patients developed bronchial asthma during the follow‐up [22]. A recent study [58] showed that NEHI has a general positive, good, but not all consistent, improvement over time, and they emphasize the importance of needing future studies to better identify different prognosis trajectories in NEHI patients. In spite of the possibility that NEHI may have long‐term adverse effects on lung function and lifelong complications, it remains unclear whether NEHI is linked to the idiopathic proliferation of pulmonary NECs in adults [32].

### 6.3 Psychosocial Impact of NEHI on Patients and Their Caregivers

NEHI may cause anxiety and distress, even depression, in patients and their caregivers due to the persistence of symptoms, delay in diagnosis, unpredictable course of the disease, and lack of effective treatment. In addition, navigating the healthcare system can pose formidable challenges for families having kids affected by NEHI, particularly when confronted with the complexities of diagnosis and treatment. The journey often entails enduring a series of medical consultations and diagnostic tests, which can be distressing. Furthermore, any misdiagnosis along this journey can exacerbate the stress and anxiety of patients and their caregivers. In such circumstances, providing not only physical care but also robust emotional support becomes imperative. It is crucial to reassure patients and their families that while NEHI presents challenges, the majority of cases exhibit a self‐limited trajectory, and there is a lack of recorded fatalities directly attributable to NEHI. Fostering a supportive environment where patients and caregivers feel heard and understood can significantly mitigate the adverse psychosocial impact of NEHI.

## 7 Conclusion

The nonspecific nature of the clinical presentation of NEHI in children has made diagnosis challenging. In light of the increasing number of reports of NEHI in children, pediatricians should remain vigilant to prevent underdiagnosis and misdiagnosis of these cases. Clinical practitioners need to closely integrate the child's clinical presentation with the ancillary examinations when diagnosing NEHI in children and pay particular attention to the imaging features of HRCTs of the chest. Since this disease progresses slowly and does not carry a high mortality risk, it is not recommended to perform a lung tissue biopsy to confirm the diagnosis. In order to prevent excessive anxiety in the family of the patient after the diagnosis has been made, proper education and psychological guidance should be provided, along with active relief of hypoxic symptoms, management of complications, and prevention of infection. Overall, the prognosis for children with NEHI is good, but they still need to be closely monitored on a regular basis over the long term [32].

## Author Contributions

Conceptualization: Long Jin and Wen Wei. Manuscript drafting: Long Jin. Manuscript revision: Wen Wei.

## Ethics Statement

The authors have nothing to report.

## Conflicts of Interest

The authors declare no conflicts of interest.

## Data Availability

Data sharing is not applicable to this article as no new data were created or analyzed in this study.
